# A “*Candidatus* Liberibacter asiaticus”-secreted polypeptide suppresses plant immune responses in *Nicotiana benthamiana* and *Citrus sinensis*

**DOI:** 10.3389/fpls.2022.997825

**Published:** 2022-10-24

**Authors:** Pan Shen, Xueyi Li, Shimin Fu, Changyong Zhou, Xuefeng Wang

**Affiliations:** National Citrus Engineering Research Center, Citrus Research Institute, Southwest University, Chongqing, China

**Keywords:** huanglongbing (HLB), *C*Las, PCD suppressor, effector, HR, plant immunity, SDE1 (CLIBASIA_05315)

## Abstract

Citrus Huanglongbing (HLB), known as the most economically devastating disease in citrus industry, is mainly caused by phloem-restricted Gram-negative bacterium “*Candidatus* Liberibacter asiaticus” (*C*Las). To date, *C*Las is still unculturable *in vitro*, which has been dramatically delaying the research on its pathogenesis, and only few Sec-dependent effectors (SDEs) have been identified to elucidate the pathogenesis of *C*Las. Here, we confirmed that a *C*Las-secreted Sec-dependent polypeptide, namely SECP8 (CLIBASIA_05330), localized in nucleus, cytoplasm and cytoplasmic membrane, and showed remarkably higher transcript abundance in citrus than in psyllids. *Potato virus X* (PVX)-mediated transient expression assays indicated that mSECP8 (the mature form of SECP8) suppressed pro-apoptotic mouse protein BAX and *Phytophthora infestans* elicitin INF1-triggered hypersensitive response (HR) associated phenotypes, including cell death, H_2_O_2_ accumulation and callose deposition. Intriguingly, mSECP8 also inhibited SDE1 (CLIBASIA_05315)-induced water-soaked and dwarfing symptoms in *Nicotiana benthamiana*. In addition, mSECP8 can promote the susceptibility of transgenic Wanjincheng orange (*Citrus sinensis*) to *C*Las invasion and further HLB symptom development, and it contributes to the proliferation of *Xanthomonas citri* subsp. *citri* (*Xcc*). Moreover, the expression of ten immunity-related genes were significantly down-regulated in mSECP8 transgenic citrus than those in wide-type (WT) plants. Overall, we propose that mSECP8 may serve as a novel broad-spectrum suppressor of plant immunity, and provide the first evidence counteractive effect among *C*Las effectors. This study will enrich and provide new evidences for elucidating the pathogenic mechanisms of *C*Las in citrus host.

## Introduction

Huanglongbing (HLB, also referred to as citrus greening), is the most economically destructive disease of citrus (Bové, 2006; Wang, 2019). The causative agents of HLB consist of three species, namely “*Candidatus* Liberibacter asiaticus (*C*Las),” “*Ca*. L. africanus (*C*Laf),” and “*Ca*. L. americanus (*C*Lam),” which are the Gram-negative bacteria, belonging to the phloem-limited, alpha-proteobacteria (Jagoueix et al., 1994; Teixeira et al., 2005; Bové, 2006). Among them, *C*Las transmitted by Asian citrus psyllid (ACP, *Diaphorina citri*), dominantly threats citrus production because of its wide distribution and strong pathogenicity (Coletta-Filho et al., 2004; Haapalainen, 2014).

In general, host plants respond to pathogen infections through two-branched innate immune systems, namely, pathogen-associated molecular patterns (PAMP)-triggered immunity (PTI) and effector-triggered immunity (ETI), generating a “zig-zag” model in plant immune system (Jones and Dangl, 2006; Boller and Felix, 2009; Yuan et al., 2021). Many extracellular plant bacterial pathogens use a type III secretion system (T3SS) to deliver virulence-associated effectors to suppress the plant innate immunity (Yamane et al., 2004; Jones and Dangl, 2006; Wang et al., 2011; Toruño et al., 2016). However, the insect-transmitted and intracellular Gram-negative bacteria, such as *C*Las, lack T3SS, but can employ a complete Sec-dependent secretion pathway to secret effectors (Duan et al., 2009; Green and Mecsas, 2016; Wang et al., 2017). When invading into plant host cells, *C*Las induces a series of immune responses, for instance, induction of pathogenesis-related (*PR*) genes, callose deposition, reactive oxygen species (ROS) burst and enhanced level of salicylic (SA) (Wang, 2019; Pang et al., 2020; Ibanez et al., 2022).

Previous studies have confirmed that many *C*Las-associated Sec-dependent effectors (SDEs) as virulence factors can suppress or trigger plant immune responses (Jain et al., 2015, 2018; Pitino et al., 2016, 2018; Clark et al., 2018; Liu et al., 2019; Zhang et al., 2019, 2020; Pang et al., 2020; Du et al., 2021; Du J. et al., 2022; Du M. et al., 2022). For instance, non-classical secretory proteins (ncSecPs) SC2_gp095 and Lasbcp (CLIBASIA_RS00445), down-regulation the expression of *RbohB* gene associated with H_2_O_2_-mediated defense signaling in plants (Jain et al., 2015, 2018). m3875 (CLIBASIA_03875) and m4405 (CLIBASIA_04405) function as two hypersensitive response (HR)-based cell death suppressors in *Nicotiana benthamiana* (Zhang et al., 2019, 2020). SDE15 (CLIBASIA_04025) inhibits plant immunity extensively through interacting with citrus susceptibility gene *CsACD2* (*ACCELERATED CELL DEATH 2, ACD2*). Intriguingly, ten ncSecPs were identified as cell death suppressors that induce the expression of *PR-1, PR-2*, and *PR-5* in *N. benthamiana* (Du et al., 2021). Systemic acquired resistance (SAR)-related genes were modulated, and *C*Las colonization was promoted in transgenic citrus by *C*aLasSDE115 (CLIBASIA_05115) (Du M. et al., 2022). Only a few *C*Las secretory proteins trigger HR in plants. SDE1 (CLIBASIA_05315) induces strong callose deposition, cell death and starch accumulation in *N. benthamiana*, and inhibits the activities of defense genes papain-like cysteine proteases (PLCPs) in citrus (Pitino et al., 2016, 2018; Clark et al., 2018). Prophage-encoded ncSecP AGH17470 triggers PR-associated and salicylic acid (SA)-signaling pathway genes expression and then enhances the resistance of citrus to *Xanthomonas citri* subsp. *citri* (*Xcc*) (Du J. et al., 2022).

*Candidatus* Liberibacter asiaticus encodes a large number of Sec-dependent proteins with unknown functions, and currently only a few has been characterized. In this study, a Sec-dependent protein SECP8 was identified through bioinformatics analyses and *E. coli* alkaline phosphatase (PhoA) assay. Its function was characterized by transient expression in *N. benthamiana* and overexpression in Wanjincheng orange (*Citrus sinensis*). The results reveal that mSECP8 is a virulence factor by suppressing plant immunity to promote *C*Las invasion.

## Materials and methods

### Plant materials, microbial strains, and growth conditions

Wild-type (WT) *N. benthamiana* plants were grown in a growth chamber maintained at 25 ± 2°C and 16 h light/8 h dark photoperiod. Plants at the five-leaf stage were used for infiltration. The transformant *Escherichia coli* strains Mach-T1 and Rosetta-gami2 (DE3) were grown on Luria-Bertani (LB) medium at 37°C, and *Agrobacterium tumefaciens* strains GV3101, GV3101 (pJIC SA_Rep) and EHA105 were cultured on LB medium with 50 μg/mL kanamycin and 20 μg/mL rifampicin at 28°C.

### Total RNA extraction, cDNA synthesis, and quantitative PCR analysis

The collected leaf samples were stored in −80°C refrigerator. Total RNA was extracted with RNAiso Plus (Takara) reagent and its concentration was quantified. Then 1 μg total RNA was reverse transcribed with All-in-One 5 × RT MasterMix Reagent Kit (ABM) in a 20 μL reaction. The cDNA was diluted five times and then used for quantitative PCR (qPCR) assay in a 10 μL reaction, consisting of 5 μL of Blastaq™ 2 × qPCR MasterMix (ABM), 3.5 μL of DNase/RNase free water, 0.25 μL of each primer (10 μM), and 1 μL of diluted cDNA template. The PCR cycling consisted of an initial activation step at 95°C for 3 min, followed by 40 cycles of 95°C for 15 s and 60°C for 1 min. The *C*Las *gyrA* gene (*CLIBASIA_00325*) and citrus *actin* gene (XM_006464503.3) were chosen as internal controls. The relative expression assay of genes was performed with three biological and technical replicates, and calculated using the 2^–ΔΔCt^ method (Livak and Schmittgen, 2001). Primers used were detailed (Supplementary Table 1).

### DNA extraction and *Candidatus* Liberibacter asiaticus detection analysis

For *C*Las detection, total DNA was extracted from the newly emerged *C*Las-inoculated leaves using the Biospin Omni Plant Genomic DNA Extraction Kit (BioFlux). PCR was performed with 2 × Taq Master Mix (Novoprotein) in a 12 μL reaction. The qPCR ran in a 10 μL reaction, consisting of 5 μL of Blastaq™ 2 × qPCR MasterMix (ABM), 3.5 μL of DNase/RNase free water, 0.25 μL of each primer (10 μM), and 1 μL of DNA (200 ng/μL). The cycling comprised an initial activation step at 95°C for 3 min, followed by 40 cycles of 95°C for 15 s, 60°C for 15 s and 72°C for 15 s.

### Bioinformatics analysis

The SECP8 sequence was extracted from the genome of *C*Las strain Psy62 (GenBank No. CP001677.5) (Duan et al., 2009). Sequences of other *C*Las strains including AHCA1 (GenBank No. CP029348.1), YNJS7C (GenBank No. QXDO00000000.1), Gxpsy (GenBank No. CP004005.1), A4 (GenBank No. CP010804.1), YCPsy (GenBank No. LIIM00000000.1), JXGC (GenBank No. CP019958.1), and Ishi-1 (GenBank No. AP014595.1) were also downloaded from NCBI^1^. The signal peptide (SP) and the transmembrane domain were predicted with SignalP 3.0 using neural networks (NN) method (Bendtsen et al., 2004) and TMHMM v2.0 (Krogh et al., 2001), respectively. DNAMAN software was used for sequence alignment.

### 3,3′-diaminobenzidine trypan-blue and aniline-blue staining assays

For symptoms visualization, the infiltrated leaves were immersed in 95% ethanol (v/v) for 48 h at 37°C until the leaves became clear.

For H_2_O_2_ accumulation assay, the infiltrated leaves were sampled and soaked in the 3,3′-Diaminobenzidine (DAB) staining buffer (pH 3.0; 1 mg/mL DAB, 0.05% Tween20 and 10 mM Na_2_PO_4_) for 12 h in the dark (Thordal-Christensen et al., 1997; Vanacker et al., 2000). Then the leaves were transferred into boiled bleaching solution (20% acetic acid, 20% glycerin, and 60% ethanol) for discoloration.

For programmed cell death (PCD) observation, the injected leaves were soaked in the trypan-blue staining solution (0.067% w/v trypan-blue, 11.11% w/v phenol, 1 vol of glycerol, 1 vol of lactic acid, 1 vol of double distilled water, 6 vol of 95% ethanol) and boiled for 2 min. Then the samples were transferred into chloral hydrate (1.25 g/mL) and shaken at 50 rpm until leaves were fully de-stained (Heese et al., 2007).

For callose deposition assay, the plant leaves were incubated in solution (acetic/glycerin/ethanol, 1v/1v/3v) overnight until the green color faded away, then washed with 150 mM K_2_HPO_4_ for 30 min. Finally, samples were stained with aniline-blue solution (150 mM K_2_HPO_4_, 0.05% w/v aniline-blue), and the callose deposition was observed *via* fluorescence microscope (Sebastian and Adam, 2015).

### Agrobacterium-mediated transient expression assay in *Nicotiana benthamiana*

The coding sequence of mSECP8, SECP8, and SDE1 were amplified with corresponding primers (Supplementary Table 1), and cloned into *Cla*I/*Sal*I linearized pGR107 vector (a *Potato virus X*-based expression vector, PVX) to generate experimental recombinant plasmids. These constructs were then transferred into *A. tumefaciens* GV3101 (pJIC SA_Rep). The PCD suppression assay was performed as described previously (Wang et al., 2011). Briefly, inoculation buffer (10 mM MES, pH 5.6, 10 mM MgCl_2_, and 100 μM acetosyringone [AS]) or *A. tumefaciens* cells harboring PVX-SECP8, PVX-mSECP8 and PVX-eGFP (negative control) with OD_600_ adjusting to 1.0 were firstly infiltrated into the newly expanded leaves of *N. benthamiana* plants. After 24 h, *A. tumefaciens*-mediated PVX-BAX, PVX-INF1 or PVX-SDE1 was infiltrated into the same area of leaves. At 48 h post inoculation (hpi), partial infiltrated leaves were used for H_2_O_2_ accumulation assay, while the remaining leaves were used for observation of the cell death phenotypes up to 7 days post inoculation (dpi). The suppression effects of SECP8 on SDE1 transient expression plants were evaluated at 15 dpi.

### Alkaline phosphatase assay

The signal peptide sequence of SECP8 (SECP8SP) was amplified with the specific primers (Supplementary Table 1) and cloned into pET-mphoA vector. The recombinant plasmids pET-mphoA (negative control), pET-phoA (positive control) and pET-SECP8SP-mphoA were transformed into *E. coli* Rosetta-gami2 (DE3), then the *E. coli* cells were grown at 37°C for 12 h on indicator LB solid medium (with 90 μg/mL 5-bromo-4-chloro-3-indolyl phosphate [BCIP], 200 μg/mL isopropyl-β-D-thiogalactopyranoside [IPTG], 75 mM Na_2_HPO_4_, and 50 μg/mL Kanamycin) (Liu et al., 2019). Transformants changed to blue were considered with PhoA activity, in contrast to the white transformants without PhoA activity.

### Subcellular localization of mSECP8 in *Nicotiana benthamiana*

The coding sequence of mSECP8 was amplified with specific primers (Supplementary Table 1) and ligated into *Kpn*I/*Xba*I double-digested pCAMBLIA1300-35S-eGFP vector. The infiltration of the experimental recombinants into *N. benthamiana* leaves was mediated by *A. tumefaciens* EHA105, meanwhile, plasma membrane marker pm-rk *CD3*-1007 (mcherry) (Nelson et al., 2007) and nuclear marker H_2_B-RFP (Liu et al., 2019) were co-infiltrated. The inoculated leaves were collected after 2 dpi and observed with confocal laser scanning microscopy.

### Genetic transformation of Wanjincheng orange (*Citrus sinensis*)

The coding region of mSECP8 was obtained by PCR with the designated primers (Supplementary Table 1), subsequently inserted into *BamH*I/*EcoR*I-digested pCAMBIA-GN-35S-MCS-NOS (pLGN) vector. To obtain the transgenic plants, the epicotyl explants of Wanjincheng orange were used for *A. tumefaciens*-mediated transformation (Peng et al., 2015; Zou et al., 2017). The successful transgenic lines were identified with both GUS histochemical staining and reverse transcription-PCR (RT-PCR) assays. All transgenic and WT control plants were grafted onto *Poncirus trifoliata* for following evaluation.

### The susceptibility evaluations of transgenic citrus to huanglongbing and canker

Half-year-old transgenic and WT Wanjincheng plants were graft inoculated with *C*Las positive buds by qPCR verification. The establishment of *C*Las on transgenic plants was firstly assayed at 40 dpi by both PCR and qPCR (Jagoueix et al., 1996; Li et al., 2006), with the *cytochrome oxidase* gene of *Citrus sinensis* as internal control, then detected monthly afterward. All samples were performed with three biological and technical replicates. The primers used were listed in Supplementary Table 1.

To assess the susceptibility of transgenic Wanjincheng plants to *Xcc*, the *in vitro* pinprick inoculation was carried out as previously described (Peng et al., 2015, 2017; Zou et al., 2021). Four leaves per transgenic line and WT control were tested, and per leaf contained six infected sites of which each comprised six punctures in 0.5 mm diameter by a needle, 1 μL bacterial *Xcc* suspension (2.5 × 10^8^ CFU/mL) was subsequently dropped onto each puncture site. Symptom Photographs were taken at 12 dpi, and the lesion sizes were assessed by ImageJ software. For each transgenic lines and WT plants, the disease indexes of 36 punctures per leave were calculated depending on the following rating index: 1, < 0.75 mm^2^ (lesion size); 2, 0.75–1.25 mm^2^; 3, 1.25–1.75 mm^2^; 4, 1.75–2.25 mm^2^; 5, 2.25–2.75 mm^2^; 6, 2.75–3.25 mm^2^; 7, >3.25 mm^2^. The disease index (DI,%), indicating the level of susceptibility to *Xcc*, was calculated using the formula (Peng et al., 2017):


$$DI=\frac{\sum \left[numberofeachindex\times thecorrespondingindex\right]}{\left[numberoflesions\times themaxindex\right]}\times 100$$

At 10 dpi, to count the number of *Xcc* colonies, the individual disease spots of inoculated leaves were detached using a 5-mm diameter size hole puncher. Three leaf discs were collected together and firstly ground in 200 μL sterile double distilled water (ddH_2_O), then added another 800 μL ddH_2_O. The suspension was mixed by 10 times gradient dilution (10^–3^ to 10^–5^) and then plated on LB medium. After incubation at 28°C for 48 h, *Xcc* colonies were counted, and the number of bacterial cells (CFU) per square centimeter of leaf tissue was calculated. Each specific dilution contained three replicates and the test was repeated three times. The bacterial strain *Xcc-29-1* used in this examination was provided by Professor Gongyou Chen (School of Agriculture and Biology, Shanghai Jiao Tong University, Shanghai, China).

## Results

### SECP8 encodes a conserved sec-delivered polypeptide with significantly higher expression level in citrus than in psyllids

**FIGURE 1:**
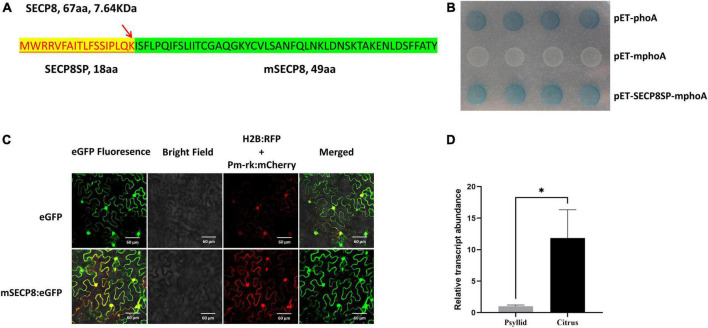
*In silico* analysis and molecular biological characteristics of SECP8. **(A)** Analysis of SECP8 amino acid sequence. Amino acid sequence of SECP8 (67 aa, 7.64 KDa) with N-terminal signal peptide (SECP8SP, highlight in yellow), red arrow indicated the cleavage site between 18 and 19 amino acid positions (LQK-IS), SECP8 without the signal peptide SECP8SP was named mSECP8 (49 aa). **(B)** *SECP8* encoded a Sec-delivered protein. PhoA assay was used for validating the secretion of *SECP8* in *Escherichia coli, E. coli* cells harboring the SECP8SP-mPhoA fusion protein turned blue which were similar to PhoA protein (positive control), while negative control mPhoA (without the SP) protein exhibited white on indicator LB medium. It was photographed after 12 h incubation at 37°C. **(C)** mSECP8 was localized in multiple subcellular compartments of *Nicotiana benthamiana*. Subcellular localization of mSECP8:eGFP fusion protein was observed *via* confocal microscopy with the following parameters: eGFP, 488 nm laser wavelength and 510 nm emission wavelength; H2B and Pm-rk, 561 nm laser and 583 nm emission filters. A histone 2B (H2B) fusion with the red fluorescent protein (RFP) and pm-rk *CD3-1007* (mcherry) were used as nuclear and plasma membrane location marker, respectively. The samples were collected at 2 dpi. Scale bars represent 60 μm. **(D)** Relative expression of SECP8 in citrus and psyllids. Transcript abundance of SECP8 was normalized against its expression in *C*las-infected Wanjincheng orange and psyllids by RT-qPCR. Bars represented the standard error (SE) of the means, *CLasgyrA* (*CLIBASIA_00325*) was used as an internal reference. The asterisks indicate significant difference (*p < 0.05, determined by Student’s *t*-test). The experiment comprised at least three independent biological replicates and three technical replicates.

*In silico* analysis of SECP8 (CLIBASIA_05330) indicated that the polypeptide is comprised of 67 amino acids (aa) with an 18-aa predicted signal peptide (namely SECP8SP) in the N-terminal (Figure 1A and Supplementary Figure 1B), and SECP8 was predicted without transmembrane domain (Supplementary Figure 1C). A BLASTn search from the GenBank database revealed that *SECP8* encoded a hypothetical protein with 100% similarity to the sequence from all available *C*Las strains, among them, the *SECP8* homologies from eight representative strains were employed for sequence alignments (Supplementary Figure 1A), while the homology of SECP8 could not be found in *C*Laf and *C*Lam genomes, suggesting *SECP8* is unique in *C*Las strains.

Through phoA assay, the *E. coli* (Rosetta-gami2 DE3 strain) carrying the fusion protein SECP8SP-mphoA turned blue after 12 h of incubation on indicator LB medium as the positive control, while the negative control construct remained white (Figure 1B). Taken together, the phoA assays along with bioinformatics analysis indicated SECP8 as a typical Sec-delivered polypeptide.

To determine the subcellular localization of SECP8, the infiltrated leaves were collected and observed by confocal fluorescence microscopy. Similar to the distribution of eGFP control, the green fluorescence signals of mSECP8 was observed in the nucleus, cytoplasm and cytoplasmic membrane as the membrane and nucleus markers (Figure 1C).

The expression pattern of *mSECP8* was further analyzed in *C*Las-infected citrus and psyllids by reverse transcription-quantitative PCR (RT-qPCR). The transcript level of *SECP8* was significantly higher (∼12-fold) in citrus host than in psyllids vectors (Figure 1D), suggesting that SECP8 may play a more important role *in planta*.

### mSECP8 suppresses BAX- and INF1-triggered hypersensitive cell death in *Nicotiana benthamiana*

**FIGURE 2:**
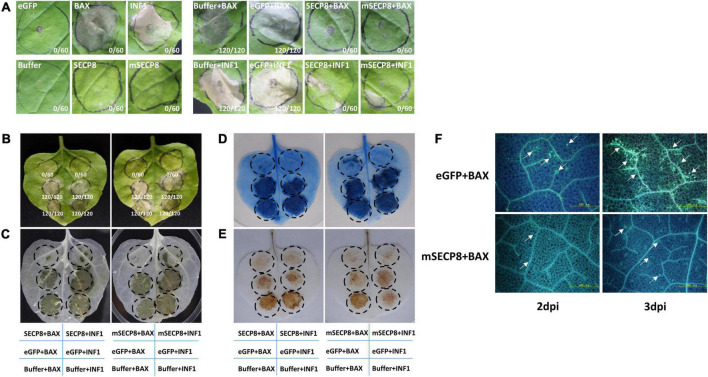
Cell death, H_2_O_2_ accumulation and callose deposition suppressed by mSECP8. **(A,B)** The SECP8 or mSECP8 suppressed the hypersensitive cell death triggered by BAX and INF1 in *Nicotiana benthamiana*. The *Agrobacterium* GV3101 (pJIC SA_Rep) strain harboring *SECP8, mSECP8, eGFP, BAX*, and *INF1*, or inoculation buffer were infiltrated into the leaves of *N. benthamiana*, either alone or followed 24 h later with GV3101 carrying *BAX* or *INF1* within the regions marked by the dashed circle. Numbers in the photos, for instance 0/60, indicated that no infiltration spot exhibited cell death out of 60 infiltration spots. The leaves were harvested at 7 days after infiltration *BAX* and *INF1*, followed by **(C)** decolorization with ethanol and **(D)** trypan-blue staining (blue-stained tissues indicated dead cells). **(E)** SECP8 or mSECP8 inhibited burst of H_2_O_2_ triggered by BAX and INF1. Samples were detached at 2 days after transient expression of BAX and INF1 for 3,3′-diaminobenzidine (DAB) staining. **(F)** SECP8 or mSECP8 suppressed accumulation of callose caused by BAX. Leaves inoculated with BAX at 2 and 3 days later were sampled for aniline-blue staining. Scale bars represent 500 μm.

The mouse BAX and the *P. infestans* INF1 are well-known as cell death elicitors and widely applied to identify the PCD suppressor of pathogens (Kamoun et al., 1998; Lacomme and Cruz, 1999). Therefore, the effect of mSECP8 on PCD suppression was evaluated with the strategy. At 7 dpi, both SECP8 and mSECP8 suppressed BAX- and INF1-induced cell death in *N. benthamiana*, whereas the phenomenon has not been observed in eGFP and buffer infiltration (Figures 2A,B). The result was confirmed by ethanol decolorization and trypan-blue staining assays (Figures 2C,D).

To further explore the role of mSECP8 on cell death suppression, the accumulation of H_2_O_2_ as a marker of plant cell death was assayed in *N. benthamiana* leaves by DAB staining. We found that mSECP8 significantly inhibited H_2_O_2_ burst triggered by both BAX- and INF1 (Figure 2E). In contrast, neither eGFP nor the infiltration buffer interfered with H_2_O_2_ accumulation.

Callose deposition as another indicator during plant immunity responses was examined. At 2∼3 dpi, mSECP8 dramatically reduced BAX-triggered callose deposition compared to eGFP control, particularly at 3 dpi (Figure 2F). Herein, these results clearly demonstrate that SECP8 is a PCD suppressor.

### mSECP8 inhibits SDE1 induced water-soaked necrosis and plant dwarfing in *Nicotiana benthamiana*

**FIGURE 3:**
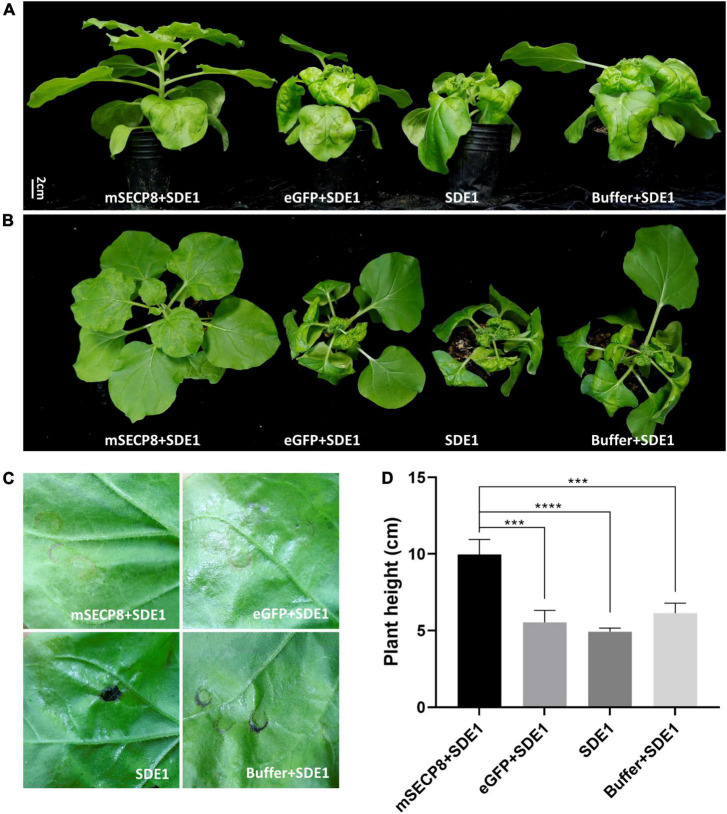
mSECP8 inhibited water-soaked necrosis and dwarfing phenotype induced by SDE1. *Nicotiana benthamiana* leaves were infiltrated with *A. tumefaciens* harboring *mSECP8, eGFP* or with buffer followed 24 h later by *Agrobacterium tumefaciens* carrying *SDE1*. **(A,B)** Photos were taken at 15 days for plant phenotypes and **(C)** 4 days for inoculated-leaves in *N. benthamiana*, respectively. **(D)** The height of *N. benthamiana*-inoculated plants at 15 dpi. The asterisks indicate significant difference (***p < 0.001, ****p < 0.0001, determined by one-way ANOVA with Dunnett’s test). Scale bar represents 2 cm in **(A)**. The experiment contains at least three independent biological replicates.

Previously, SDE1 (CLIBASIA_05315) was identified as a *C*Las Sec-dependent effector and induced water-soaked necrosis in *N. benthamiana* at 4 dpi (Pitino et al., 2016, 2018). Then we examined whether the PCD suppressor SECP8 interferes SDE1 function. We observed that SDE1 induced water-soaked necrosis was present at injection areas at 4 dpi (Figure 3C) and the plants even displayed severe necrosis, dwarfing, leaf curling and shrinking phenotypes at 15 dpi (Figures 3A,B). Similar symptoms were also observed in *N. benthamiana* infected with *eGFP* + *SDE1* and buffer + *SDE1*. However, SDE1 induced phenotypes were not observed in plants by co-infiltrated with *mSECP8* (Figures 3A,B,D). Additionally, no *SDE1*-associated phenotypes appeared on eGFP or mSECP8 single infiltration plants (Supplementary Figures 2A,B). Therefore, these results indicate that PCD suppressor mSECP8 inhibits SDE1-mediated necrosis and dwarfing in *N. benthamiana*.

### mSECP8 enhances the susceptibility of transgenic citrus to both *Candidatus* Liberibacter asiaticus and *Xanthomonas citri* subsp. *citri*

**FIGURE 4:**
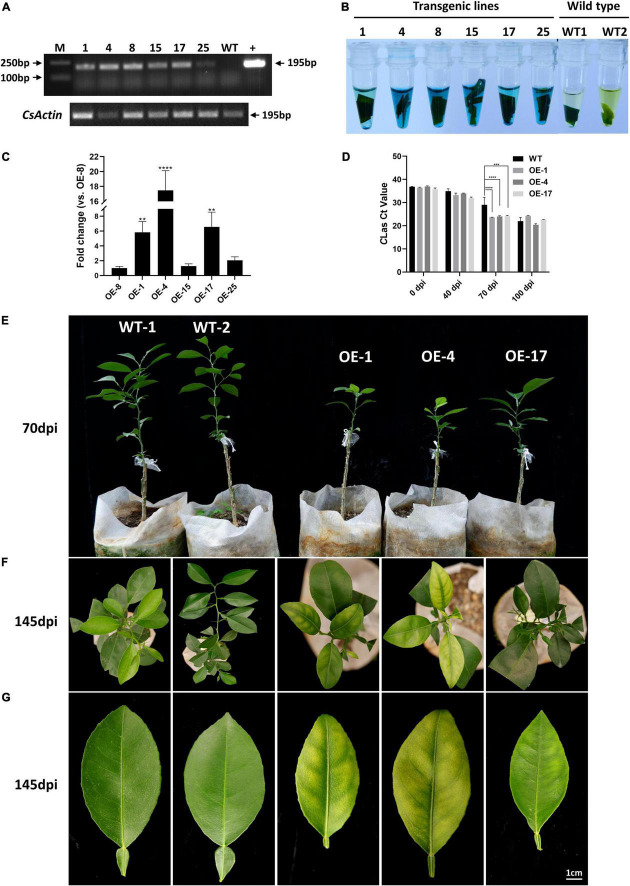
*mSECP8*-transgenic Wanjincheng orange plants were more susceptible to *C*Las. **(A–C)** Identification of *mSECP8*-transgenic plants. M, DL 2,000 DNA marker; 1, 4, 8, 15, 17, and 25 represented different *SECP8*-transgenic lines; WT1 and WT2, wild type of Wanjincheng plants; + , pLGN-mSECP8 plasmid. **(A)** A 195 bp fragment was detected by RT-PCR using pLGN-mSECP8-F/R primer pairs (Supplementary Table 1), the *actin* gene of *citrus sinensis* (*CsActin*) was used as internal reference. **(B)** Leaves of transgenic plants were used for GUS staining, and the blue-stained indicated positive plants. **(C)** SECP8 relative expression level in OE-1, OE-4, and OE-15, OE-17, and OE-25 compared with that in OE-8 was determined by RT-qPCR, the *CsActin* gene was used as internal reference. The asterisks indicate significant difference (**p < 0.01, ****p < 0.0001, determined by one-way ANOVA with Dunnett’s test). **(D)** The *C*las titers in the *mSECP8*-transgenic citrus and WT control citrus were determined by qPCR at 0, 40, 70, and 100 dpi with *C*Las, the *cytochrome oxidase* gene *of citrus sinensis* (*CsCOX*) was chosen as internal control. Each Ct value was represented by means ± SE (*n*_OE–mSECP8_ = 3, *n*_WT_ = 3). Asterisks represent significant differences in the *C*las titer of 0, 40, 70, and 100 dpi between *mSECP8*-transgenic citrus and WT control by two-way ANOVA with Tukey’s test (***P < 0.001, and ****P < 0.0001). **(E)** Plants phenotype obtained at 70 dpi with *C*las *via* branch and budding grafting. **(F)** Top view of *C*Las-infected WT and transgenic Wanjincheng plants at 145 dpi, and **(G)** symptoms of single leave detached from the contemporaneous plants. Scale bar represents 1 cm.

To evaluate the role of mSECP8 on *C*Las colonization, transgenic citrus plants were generated and six lines (OE-1, OE-4, OE-8, OE-15, OE-17, and OE-25) were verified by both RT-PCR and GUS histochemical staining assay (Figures 4A,B). By comparing to line OE-8, the three lines (OE-1, OE-4, and OE-17) with greater transcript abundance were graft inoculated with *C*Las for further susceptibility evaluation (Figure 4C). *C*Las titers in OE-1, OE-4, and OE-17 lines were higher than that of WT plants at 40 dpi, especially at 70 dpi (Figure 4D). Nevertheless, *C*Las titers in WT plants got close to that in transgenic plants at 100 dpi (Figure 4D). From 70 to 145 dpi, *C*Las associated chlorosis or mottled yellow symptoms were observed on the leaves of transgenic plants, and these transgenic lines were significantly dwarfing compared with WT controls (Figures 4E–G), while transgenic treatments and WT control showed no phenotype differences without *C*Las inoculation (Supplementary Figures 3A–C). These results suggest that *mSECP8* contributes to *C*Las early proliferation and symptom development in transgenic citrus plants.

To observe whether *mSECP8* enhanced susceptibility of transgenic plants to other pathogens, transgenic citrus leaves were inoculated using *Xcc*. Compared to WT plant, the lesion size (LZ) increased by 147.3∼289.6% (Supplementary Figure 4A), and the disease index (DI) increased from 22.9 to 53.2∼81.8% (Supplementary Figure 4B), and significantly more colony-forming units (CFUs) were observed in transgenic plants (Supplementary Figure 4C). Furthermore, leaves of *Xcc*-inoculated *mSECP8*-overexpressing plants exhibited remarkably earlier and larger pustule eruptions than those of control plants (Supplementary Figure 4D).

### The expression level of the defense-related genes are remarkably decreased in mSECP8-transgenic citrus

**FIGURE 5:**
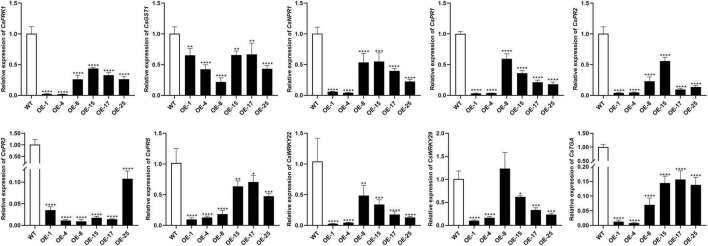
mSECP8 down-regulated the expression of defense-related genes in transgenic citrus. Transcript level of *CsFRK1, CsGST1, CsNPR1, CsPR1, CsPR2, CsPR3, CsPR5, CsWRKY22, CsWRKY29*, and *CsTGA* were significantly lower in *mSECP8*-transgenic than that in WT Wanjincheng plants. The *CsActin* gene was used as an endogenous control. The asterisks indicate significant difference (*P < 0.05, **p < 0.01, ***P < 0.001 and ****P < 0.0001, one-way ANOVA with Dunnett’s test). The experiment comprised at least three independent biological replicates and three technical replicates.

To further investigate how mSECP8 interferes with plant defense responses, the expression of 10 immunity-related genes were analyzed. The results showed that the expression level of all the SAR-related (*CsPR1, CsPR2, CsPR3*, and *CsPR5*) and PTI marker (*CsFRK1, CsGST1, CsWRKY22*, and *CsWRKY29*) genes were significantly reduced in transgenic plants (Figure 5). After challenging with *C*Las, similar results were exhibited in OE-mSECP8 plants (Supplementary Figure 5), indicating that mSECP8 may suppress the plants defense responses through interrupting SAR- and PTI-mediated pathways.

## Discussion

In the host-pathogen arms race, on one hand, to prevent the spread of pathogen, host plants defense against pathogens by PTI and ETI with HR-associated PCD; on the other hand, to invade the host plants successfully, pathogens including bacteria, fungi and oomycetes evolve various effectors as virulence factors to suppress PTI or even ETI (Nimchuk et al., 2003; Jones and Dangl, 2006; Coll et al., 2011; Nguyen et al., 2021; Yuan et al., 2021). HR suppression of plant immune system plays a leading role in pathogenic virulence strategy. As is well known, numerous pathogens including bacteria, fungi, and oomycetes colonize extracellularly and suppress host HR by T3SS-secreted or Sec pathway-dependent effectors (Boller and Felix, 2009; Segers and Anné, 2011; Rocafort et al., 2020; Yuan et al., 2021).

PCD plays critical roles in plant immunity upon pathogen infection at early stage (Coll et al., 2011). By contrast, the pathogens secrete effectors to suppress cell death to facilitate their successful infection. *C*Las encodes a large number of Sec-dependent secretory proteins with unknown functions and a few have been characterized, but with only three, SDE1, m460, and AGH17470 identified as PCD elicitors, while others functioning as suppressors (Pitino et al., 2016, 2018; Liu et al., 2019; Du J. et al., 2022). In our study, mSECP8 was also identified as a suppressor by suppressing BAX- and INF-triggered PCD syndromes. It is noteworthy that mSECP8 interfered with SDE1-mediated water-soaked necrosis, the plant dwarfing and flowering delay (data not shown) in *N. benthamiana*. Previously, *C*Las effector SDE15 was verified as a broad-spectrum suppressor to inhibit PCD elicited by *XccA^w^* and *Xanthomonas vesicatoria* effector AvrBsT (Pang et al., 2020). Both SDE1 and mSECP8 play critical roles in citrus host and potentially target multiple host proteins due to their higher mRNA transcriptional levels in citrus than in psyllids with diverse subcellular localization. SDE1 is systematically distributed and has been demonstrated to interact with host PLCP proteins in reducing target activity to promote *C*Las infection (Clark et al., 2018). Therefore, mSECP8 is speculated as a potential broad-spectrum suppressor of plant immunity, and potentially interacting with *C*Las elicitor SDE1 to co-manipulate plant immune responses. The counteractive effect of the two effectors could assist *C*Las escaping from host immunity, resulting in long latency and slowly developed symptoms of HLB. However, the molecular mechanisms of how mSECP8 interfering with SDE1 remains to be determined.

Systemic acquired resistance is highly associated with plant defense against bacterial pathogens (Durrant and Dong, 2004). In *C*Las-citrus interaction system, the expression of SAR-related genes (*CsPR1, CsPR2, CsPR3*, and *CsPR5*) and PTI marker genes (*CsFRK1, CsGST1, CsWRKY22*, and *CsWRKY29*) were remarkably down-regulated in transgenic plants expressing *SDE15* by challenged with *XccA^w^*. Similarly, the transcript abundance of *WRKY* and *PR* genes was reduced in *mSDE115*-overexpressing citrus (Pang et al., 2020; Du M. et al., 2022). Whereas the expression of *PR*- and SA-related genes were significantly induced in *AGH17470*-transgenic citrus (Du J. et al., 2022). Intriguingly, 10 non-classic SecPs triggered the overexpression of *NbPR1, NbPR2*, and *NbPR5* genes (Du et al., 2021). Herein, mSECP8 potentially promoted *C*Las infection *via* repression of SAR- and PTI-mediated defenses, but the detailed mechanisms of how mSECP8 suppress plant immunity remains to be addressed through finding its host interacting targets.

Conclusively, we identified the *C*Las-secreted Sec-dependent effector SECP8 as a broad-spectrum suppressor by inhibiting elicitor-triggered PCD in *N. benthamiana* and SDE1-induced phenotypes, and thereby provide the first evidence of counteractive effect of *C*Las suppressors and elicitors. Through susceptibility assessment of transgenic plants to *C*Las and *Xcc*, it revealed that mSECP8 may be involved in suppression of the plant immunity through manipulating SAR- and PTI-mediated defenses to facilitate pathogen infection. The present findings illustrate the role of SECP8 in pathogenicity of *C*Las and may shed new insight in *C*Las-citrus interactions.

## Data availability statement

The datasets presented in this study can be found in online repositories. The names of the repository/repositories and accession number(s) can be found in the article/Supplementary material.

## Author contributions

PS, XW, and CZ designed the experiments. PS and XL performed the experiments. PS analyzed the data and wrote the manuscript. SF, XW, and CZ revised and polished the manuscript. All authors contributed to the article and approved the submitted version.

## Footnotes

1
https://www.ncbi.nlm.nih.gov/genome/browse/#!/prokaryotes/1750/

## Supplementary material

Supplementary Figure 1 Bioinformatics features of SECP8. **(A)** The nucleotide sequence alignments among SECP8 (CLIBASIA_05330) and the homologies of other *C*Las typical strains from United States, China, and Japan. **(B)** SP and **(C)** transmembrane domain prediction of SECP8.

Supplementary Figure 2 No plant phenotype differences were induced by *eGFP* or *mSECP8*. **(A,B)** Photos were taken at 15 dpi, and scale bar represents 2 cm in **(A)**.

Supplementary Figure 3 Phenotype comparison between WT and overexpressing (OE)-*mSECP8* citrus plants. **(A–C)** One-year old plants and corresponding detached leaves were exhibited. Scale bar represents 1 cm in **(C)**.

Supplementary Figure 4 *mSECP8*-transgenic Wanjincheng orange plants were more susceptible to *Xcc*. **(A–C)** The susceptibility evaluation of *mSECP8*-transgenic plants to *Xcc*. **(A)** Lesion size (mm^2^) was analyzed using imageJ software, followed by the calculation of **(B)** disease index (DI,%) and **(C)** colony numbers (CFU/cm^2^). Each value was represented by means ± SE (*n*_OE–mSECP8_ = 3, *n*_WT_ = 3). Asterisks represent significant differences between *mSECP8*-transgenic lines and WT control by one-way ANOVA with Dunnett’s test (^****^*P* < 0.0001). CFU, colony-forming units. **(D)** At 12 dpi, disease spots were exhibited after inoculation with *Xcc* cells at a concentration of 2.5×10^8^ CFU/mL using pinprick inoculation method, scale bar represents 1 cm. The experiment comprised at least three independent biological replicates and three technical replicates.

Supplementary Figure 5 mSECP8 reduced the transcript abundance of immunity-associated genes in *C*Las-infected transgenic citrus. Expression level of *CsFRK1, CsGST1, CsPR1, CsPR2, CsPR3, CsPR5, CsWRKY22, CsWRKY29*, and *CsTGA* were significantly decreased in mSECP8-overexpressing plants. The *CsActin* was chosen as an endogenous reference. The asterisks indicate significant difference (**P* < 0.05, ^**^*p* < 0.01, one-way ANOVA with Dunnett’s test). The experiment comprised at least three independent biological replicates and three technical replicates.

Supplementary Table 1 Primers used in this study.
