# Supplementary figures and images for: A “Candidatus Liberibacter asiaticus”-secreted polypeptide suppresses plant immune responses in Nicotiana benthamiana and Citrus sinensis
Source: Front Plant Sci. 2022 Oct 24;13:997825. doi: 10.3389/fpls.2022.997825 (PMC9638108; doi:10.3389/fpls.2022.997825)

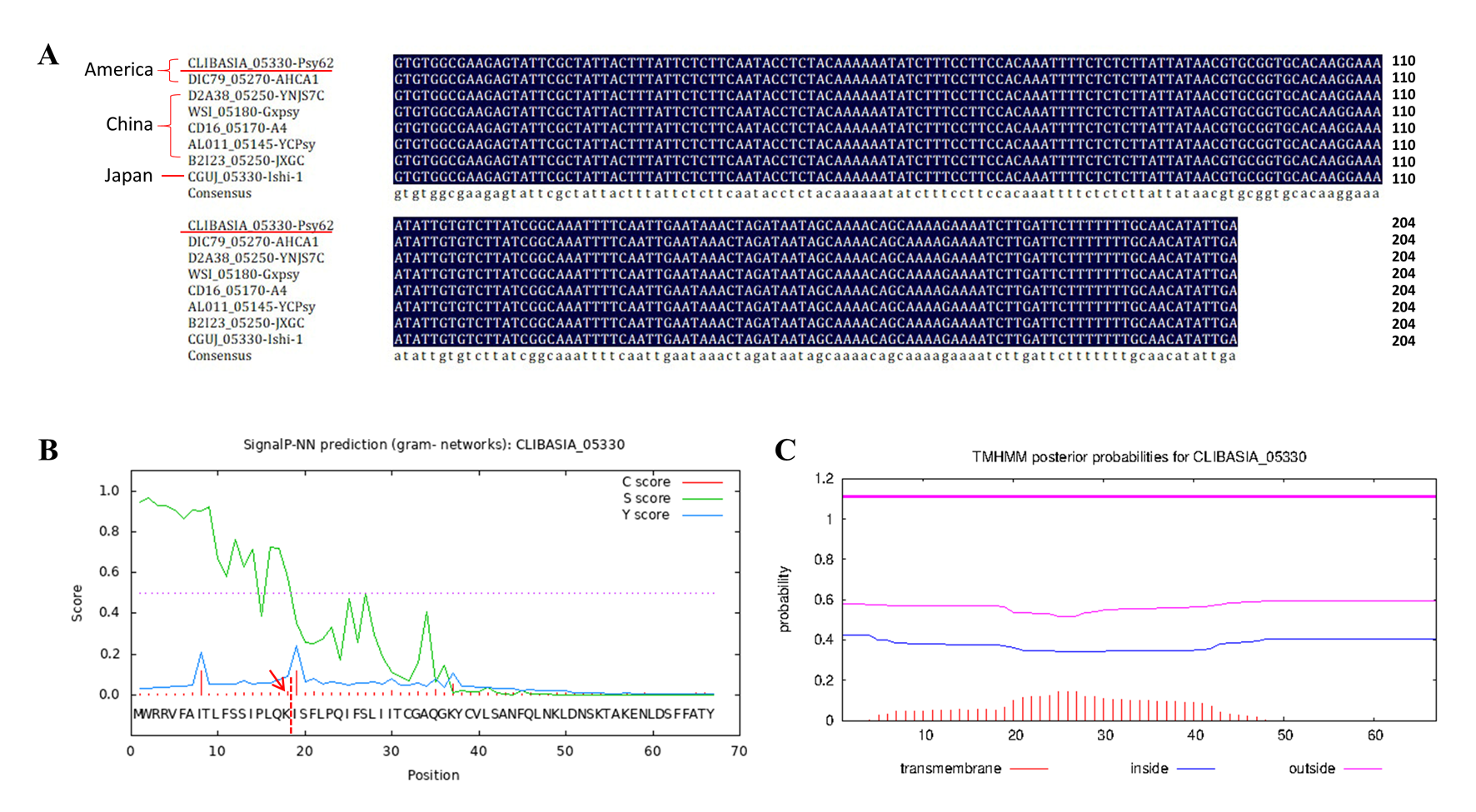

Supplement: Supplementary Figure 1 — Bioinformatics features of SECP8. (A) The nucleotide sequence alignments among SECP8 (CLIBASIA_05330) and the homologies of other CLas typical strains from United States, China, and Japan. (B) SP and (C) transmembrane domain prediction of SECP8. [file Image_1.TIF]

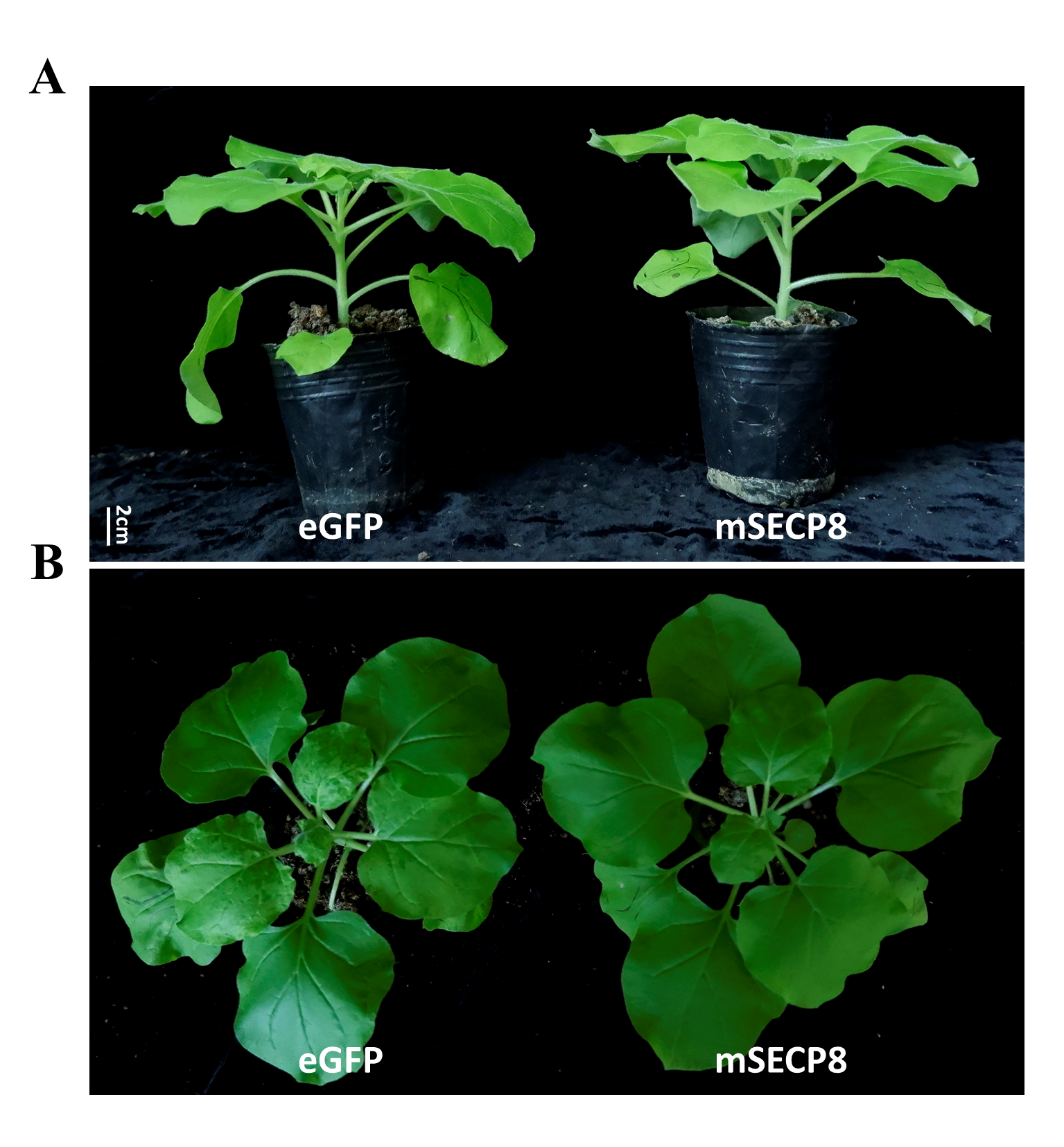

Supplement: Supplementary Figure 2 — No plant phenotype differences were induced by eGFP or mSECP8. (A,B) Photos were taken at 15 dpi, and scale bar represents 2 cm in (A). [file Image_2.TIF]

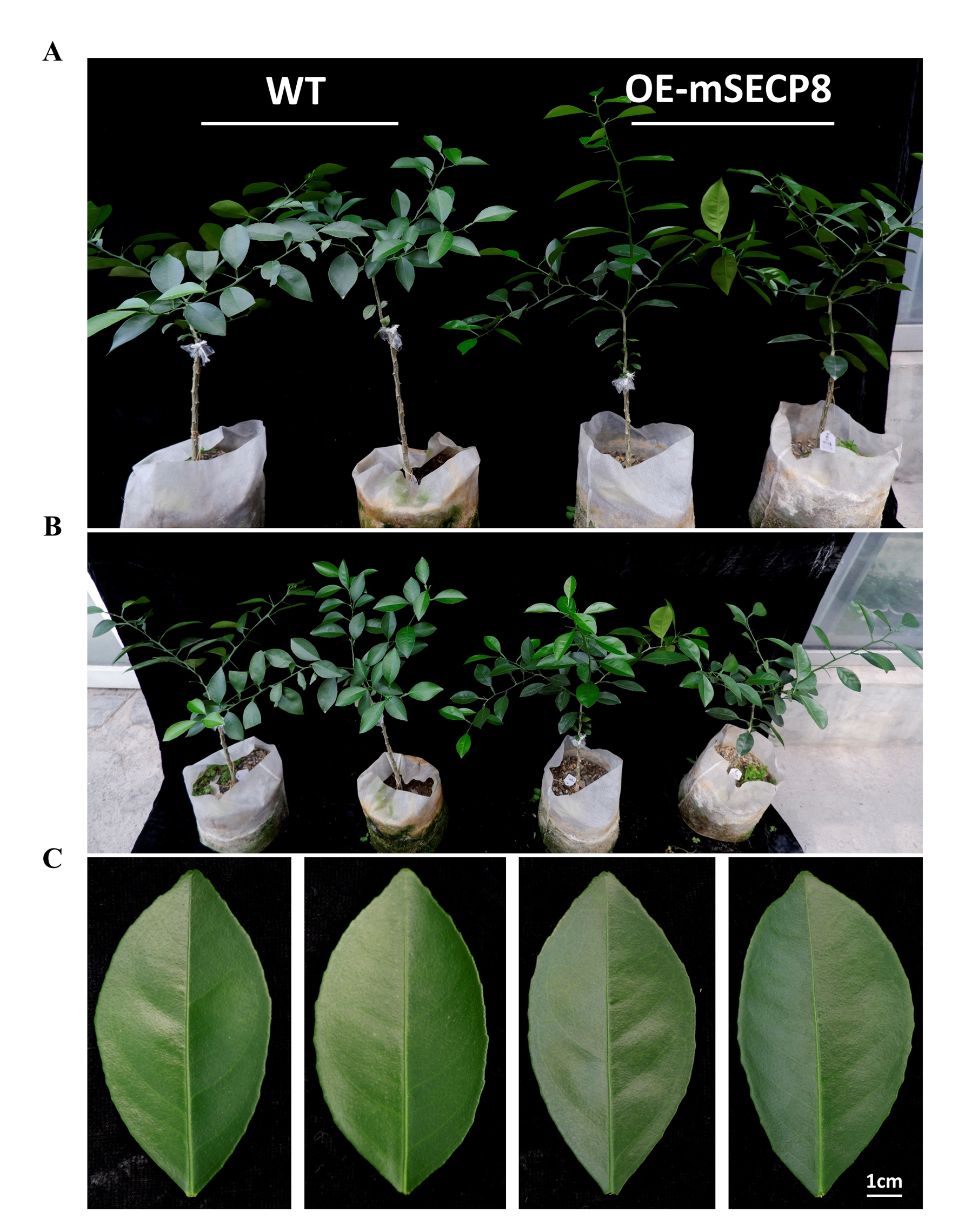

Supplement: Supplementary Figure 3 — Phenotype comparison between WT and overexpressing (OE)-mSECP8 citrus plants. (A–C) One-year old plants and corresponding detached leaves were exhibited. Scale bar represents 1 cm in (C). [file Image_3.TIF]

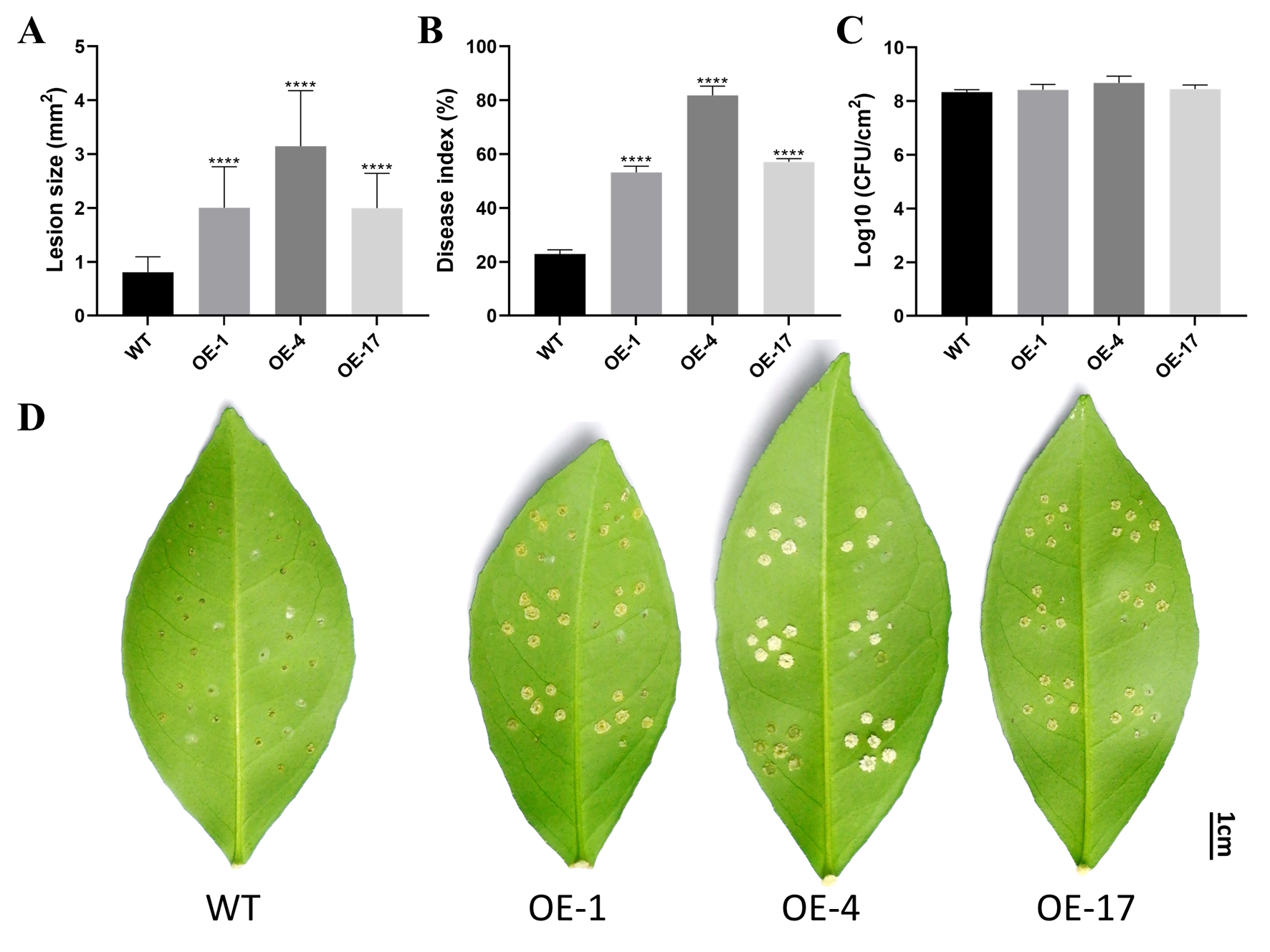

Supplement: Supplementary Figure 4 — mSECP8-transgenic Wanjincheng orange plants were more susceptible to Xcc. (A–C) The susceptibility evaluation of mSECP8-transgenic plants to Xcc. (A) Lesion size (mm2) was analyzed using imageJ software, followed by the calculation of (B) disease index (DI,%) and (C) colony numbers (CFU/cm2). Each value was represented by means ± SE (nOE–mSECP8 = 3, nWT = 3). Asterisks represent significant differences between mSECP8-transgenic lines and WT control by one-way ANOVA with Dunnett’s test (****P < 0.0001). CFU, colony-forming units. (D) At 12 dpi, disease spots were exhibited after inoculation with Xcc cells at a concentration of 2.5×10^8^ CFU/mL using pinprick inoculation method, scale bar represents 1 cm. The experiment comprised at least three independent biological replicates and three technical replicates. [file Image_4.TIF]

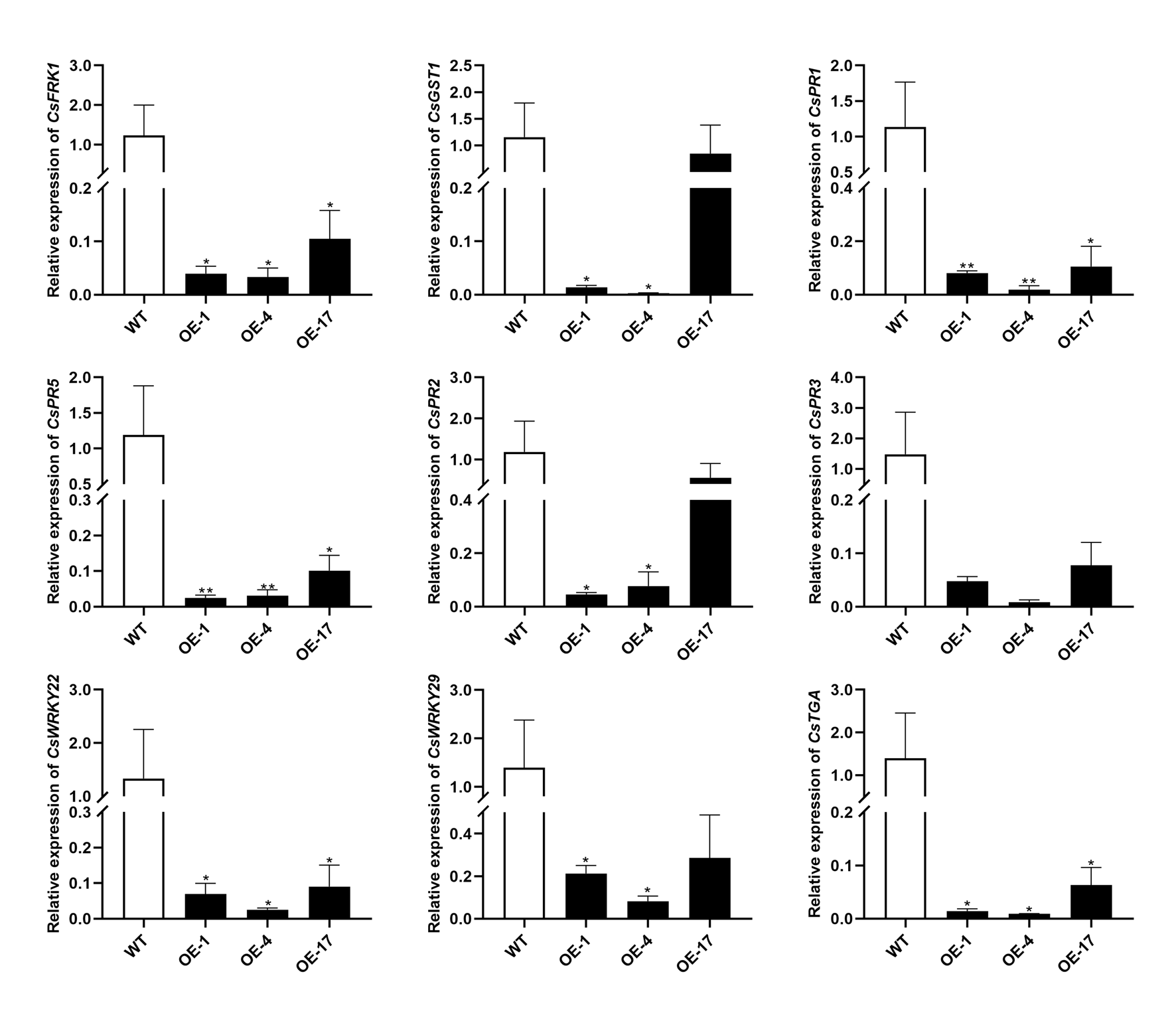

Supplement: Supplementary Figure 5 — mSECP8 reduced the transcript abundance of immunity-associated genes in CLas-infected transgenic citrus. Expression level of CsFRK1, CsGST1, CsPR1, CsPR2, CsPR3, CsPR5, CsWRKY22, CsWRKY29, and CsTGA were significantly decreased in mSECP8-overexpressing plants. The CsActin was chosen as an endogenous reference. The asterisks indicate significant difference (*P < 0.05, **p < 0.01, one-way ANOVA with Dunnett’s test). The experiment comprised at least three independent biological replicates and three technical replicates. [file Image_5.TIF]
