# Supplementary material for: A “Candidatus Liberibacter asiaticus”-secreted polypeptide suppresses plant immune responses in Nicotiana benthamiana and Citrus sinensis
Source: Front Plant Sci. 2022 Oct 24;13:997825. doi: 10.3389/fpls.2022.997825 (PMC9638108; doi:10.3389/fpls.2022.997825)
Supplement: Supplementary Table 1 — Primers used in this study. [file Table_1.docx]

**Supplementary Table 1|**Primers used in this study

| **Primer name** | **Sequence (5’ to 3’)** | **Gene Name** | **Accession number** | **Usage** | **Product size (bp)** |
| --- | --- | --- | --- | --- | --- |
| pET-SECP8SP-mphoA-F(*Nde*Ⅰ) | aaggagatatacatatgGTGTGGCGAAGAGTATTCGCTAT | *SECP8* | CLIBASIA_05330 | phoA assay | 89 |
| pEF-SECP8SP-mphoA-R (*Hind*Ⅲ) | aggcatttctggaagcttTTTTTGTAGAGGTATTGAAGAG |  |  |  |  |
| p35S-mSECP8-eGFP-F (*Kpn*Ⅰ) | cgggggacgagctcgggtaccATGATATCTTTCCTTCCACAAATTTTC | *mSECP8* | CLIBASIA_05330 | Subcellular localization assay | 192 |
| p35S-mSECP8-eGFP-R (*Xba*Ⅰ) | caccatggtgtcgactctagaATATGTTGCAAAAAAAGAATCAAGATT |  |  |  |  |
| PVX-SECP8-F (*Cla*Ⅰ) | accagctagcatcgatGTGTGGCGAAGAGTATTCGCTAT | *SECP8* | CLIBASIA_05330 | Transient expression and PCD suppression assays | 236 |
| PVX-SECP8-R (*Sal*Ⅰ) | cttatcggcggtcgacTCAATATGTTGCAAAAAAAGAATCAA |  |  |  |  |
| PVX-mSECP8-F (*Cla*Ⅰ) | accagctagcatcgatATGATATCTTTCCTTCCACAA | *mSECP8* | CLIBASIA_05330 |  | 185 |
| PVX-mSECP8-R (*Sal*Ⅰ) | cttatcggcggtcgacTCAATATGTTGCAAAAAAAGAATCAA |  |  |  |  |
| PVX-SDE1-F (*Cla*Ⅰ) | accagctagcatcgatATGGGCAGTAGTTTTGGTTGTTG | *SDE1* | CLIBASIA_05315 |  | 438 |
| PVX-SDE1-R (*Sal*Ⅰ) | cttatcggcggtcgacTCAAGACTGCTCCAACATTTTTCT |  |  |  |  |
| pLGN-mSECP8-F(*Bam*HⅠ) | ggacagggtacccggggatccATGATATCTTTCCTTCCACAAATTTTC | *mSECP8* | CLIBASIA_05330 | Transgenic experiment | 195 |
| pLGN-mSECP8-R (*Eco*RⅠ) | tctcattaaagcagggaattcTCAATATGTTGCAAAAAAAGAATCAA |  |  |  |  |
| HLB-OI1 | GCGCGTATGCAATACGAGCGGCA | *16S ribosomal RNA* | JX121094.1 | Detection of *C*Las by PCR | 1167 |
| HLB-OI2c | GCCTCGCGACTTCGCAACCCAT |  |  |  |  |
| qHLBas | TCGAGCGCGTATGCAATAC | *16S ribosomal RNA* | FJ750458.1 | Detection of *C*Las by qPCR | 75 |
| qHLBr | GCGTTATCCCGTAGAAAAAGGTAG |  |  |  |  |
| qCsCOX-F | GTATGCCACGTCGCATTCCAGA | *cytochrome oxidase subunit 1*  (*CsCOX*) | NC_037463.1 | Internal reference of *C*Las detection by qPCR | 68 |
| qCsCOX-R | GCCAAAACTGCTAAGGGCATTC |  |  |  |  |
| RT-CsActin-F | CATCCCTCAGCACCTTCC | *Citrus sinensisactin-7*  (*CsActin*) | XM_006464503.3 | Detection of genes in citrus by RT-qPCR, as internal reference | 195 |
| RT-CsActin-R | CCAACCTTAGCACTTCTCC |  |  |  |  |
| RT-*C*LasgyrA-F | GTATGGCACAGGACTGGTCT | *CLasgyrA* | CLIBASIA_00325 | Detection of genes in *C*Las by RT-qPCR, as internal reference | 173 |
| RT-*C*LasgyrA-R | GTTAGGGCGGAAATCAACAGT |  |  |  |  |
| RT-mSECP8-F | TCTCTTATTATAACGTGCGGTGC | *mSECP8* | CLIBASIA_05330 | Detection of corresponding genes by RT-qPCR | 123 |
| RT-mSECP8-R | ATATGTTGCAAAAAAAGAATCA |  |  |  |  |
| RT-CsFRK1-F | AGTGACGTGTACAGCTTTGG | *Leucine-rich repeat receptor-like protein kinase*  (*CsFRK1*) | XM_015531278.2 |  | 135 |
| RT-CsFRK1-R | TCTGATATTTCCCTCGGCAAG |  |  |  |  |
| RT-CsGST1-F | AGACATGAAGGCTGGTGAAC | *glutathione S-transferase F6*  (*CsGST1*) | XM_006479845.3 |  | 135 |
| RT-CsGST1-R | AACTCCTGGGCGATGTATTG |  |  |  |  |
| RT-CsNPR1-F | AGAGGACCCAAGTTTGAGCT | *BTB/POZ domain and ankyrin repeat-containingprotein NPR1*  (*CsNPR1*) | XM_006475416.3 |  | 146 |
| RT-CsNPR1-R | CAGGCATCATCATCCACAC |  |  |  |  |
| RT-CsPR1-F | AAATGTGGGTGAATGAGAAAGC | *pathogenesis-related protein 1-like*  (*CsPR1*) | XM_006486759.3 |  | 140 |
| RT-CsPR1-R | ATTATTGTTGCACGTCACCTTG |  |  |  |  |
| RT-CsPR2-F | TTCCACTGCCATCGAAACTG | *glucan endo-1,3-beta-glucosidase-like*  (*CsPR2*) | NM_001320062.1 |  | 73 |
| RT-CsPR2-R | GTAATCTTGTTTAAATGAGCCTCTTG |  |  |  |  |
| RT-CsPR3-F | GGCTCAAACTTCACATGAAACTAC | *basic endochitinase*  (*CsPR3*) | XM_006466989.3 |  | 113 |
| RT-CsPR3-R | GTTGACAATAATCTCCAGGGTTTC |  |  |  |  |
| RT-CsPR5-F | CACCATTGCCAATAACCCTAATG | *pathogenesis-related protein 5*  (*CsPR5*) | XM_006473389.3 |  | 148 |
| RT-CsPR5-R | GGGACAGTTACCGTTAAGATCAG |  |  |  |  |
| RT-CsWRKY22-F | AGGAAACAGGTGGAACGAAG | *WRKY transcription factor 22-like*  (*CsWRKY22*) | XM_006476427.3 |  | 148 |
| RT-CsWRKY22-R | GACCTTGAGCCTTTGACATTG |  |  |  |  |
| RT-CsWRKY29-F | ACATATACGGCAGAGCACAAC | *WRKY transcription factor 22*  (*CsWRKY29*) | XM_006491125.2 |  | 147 |
| RT-CsWRKY29-R | AGTGGGTTTGGTTGAGGAAG |  |  |  |  |
| RT-CsTGA-F | TGACAAAGAACTTCAGAAAGG | *TMV resistance protein N-like*  (*CsTGA*) | XM_015533480.2 |  | 196 |
| RT-CsTGA-R | CGTCGGTTCCACATCATAG |  |  |  |  |

Notes: The lowercase letters represent the homologous sequence with corresponding vectors,of which marked in red indicate restriction endonucleases, while the uppercase letters indicate the forward sequence of corresponding gene. The annotation information and accession number of gene were acquired from NCBI database (http://www.ncbi.nlm.nih.gov), and the gene abbreviations referred to the study were in Pang et al.(2020) and Du J et al.(2022).
